# Prevalence of Celiac Disease in Children with Idiopathic Dilated Cardiomyopathy

**Published:** 2014-07-05

**Authors:** Mozhgan Zahmatkeshan, Mahsa Fallahpoor, Hamid Amoozgar

**Affiliations:** 1 Transplant and Neonatology Research Center; 2 Department of Pediatrics; 3 Neonatology and Cardiac Research Center, Shiraz University of Medical Sciences, Shiraz, Iran.

**Keywords:** Celiac Disease, Dilated Cardiomyopathy, Children, Heart

## Abstract

***Objective:*** This study aimed to evaluate the prevalence of celiac disease (CD) in the patients with dilated cardiomyopathy (DCM). Simultaneous presentation of these two diseases has been recently reported in some studies; however, few researches have been done on children. The sooner CD is diagnosed, the better the prognosis will be, especially in the patients with a chronic disease like DCM.

***Methods:*** In this study, 82 cases were screened for CD by measuring the level of anti-body against transglutaminase (anti tTG). These cases included 41 patients with DCM labeled according to clinical evaluation and echocardiography and 41 healthy children who had been referred for routine checkup. All the patients were between 1 and 18 years old. The expired patients and those with previous diagnosis of CD were excluded from the study. Besides, the patients with positive antibody results underwent intestinal biopsy to match the serology findings with histopathology of CD in the intestine. Finally, the data were analyzed by the SPSS statistical software (v. 16) and through t-test and Pearson correlation coefficient.

***Findings:*** According to the findings, 1/41 (2.5%) DCM cases had positive tTG antibody level and negative intestinal biopsy which is classified as potential CD in the children with DCM. In addition, 7/41 (17%) patients had borderline anti body level. A direct correlation was observed between age and anti tTG level.

***Conclusion:*** It is beneficial to assess CD in DCM children with unknown cause.

## Introduction

Celiac disease (CD) is an immune-mediated disorder resulting from gluten ingestion in genetically susceptible individuals and is characterized by chronic inflammation of the small intestine^[^^1^^,^^2^^]^. Dilated cardiomyopathy (DCM) is a clinical syndrome of heart failure that is associated with impaired systolic function and left ventricular dilatation in the absence of an identifiable cause which occurs at any age^[^^3^^,^^4^^]^. The etiology of both CD and DCM is unknown. In fact, both diseases are considered to be multifactorial and some factors, such as immune-mediated disorder, genetic disorders (HLA-DQ8*, TGM2*, *PML* as up-regulated anti-angiogenic genes and to *GNA13, TGFA, ERBB2,* and *SCG2 *which present in both)*,* and carnitine deficiency, are common between the two^[^^5^^-^^9^^]^. 

 The early diagnosis of CD in patients with DCM is very important before it becomes too late to treat it with gluten-free diet, also complicated patients like DCM are usually malnourished and CD can worsen their condition^[^^10^^]^. Besides the sooner we diagnose the better is the prognosis, as gluten-free diet makes intestinal permeability better and allows adequate absorption of the drugs used in heart failure therapy. Therefore, such diet may minimize progression and delay the indication for heart transplantation. Also more than 4% of patients with myocarditis and heart failure and even arrhythmia respond to immunosuppressive therapy and gluten free diet^[^^11^^-^^13^^]^. 

 Based on what is mentioned above and considering the importance of diagnosis of such a treatable disease, like CD, in the patients with DCM and the fact that only few studies have been conducted on this issue among the children, the present study aimed to assess CD in the children with idiopathic DCM.

## Subjects and Methods

Since December 2010 until November 2013, all the children with clinical diagnosis of DCM visited at in- or out-patient service of Namazi Hospital, affiliated to Shiraz University of Medical Sciences, were included in a cross sectional case and control study. DCM was labeled by clinical manifestations and the presence of at least a left ventricle dysfunction, ejection fraction (EF) lower than 55%, ventricular dilation, and left ventricular end-diastolic diameter greater than 112% on echocardiogram^[^^4^^]^. In this study, the children between 1 and 18 years old were included to ensure gluten exposure. Also, as most of the patients with DCM are malnourished and may have problems in their immune system basically as a chronic disease and also because the kits for measuring antibodies have variations, in addition to determining whether the normal range of antibody differs in this age group, we selected 41 healthy children of the same age and sex who had been referred to the clinic for routine work ups as the control group. Then, screening test for CD was simultaneously performed for both groups. Anti-body levels of both groups were measured at the same laboratory. The patients were screened for GI symptoms and were asked about their current medication. The study was approved by Shiraz University of Medical Sciences and written informed consent was obtained from parents of all subjects.

**Serology test:**

The children were tested for anti-tissue transglutaminase (tTG) and Immunoglobulin A (IgA) antibodies. Anti-tTG of both case and control groups was measured in the same laboratory (Motahari Clinic) using Nephelometery method. IgA titering was assessed using the MININEPH kit manufactured by Binding Site Co, Germany. In addition, anti-tTG antibody level was measured by Elisa method and the kit produced by Monoband Company, USA. Anti-tTG titer was considered as positive in case it was higher than 18U/m and at borderline in case it was higher than 12 U/m according to the manufacturer’s instructions. 

 The patients also underwent serum IgA test and the acceptable level was considered according to the manufacturer’s guidelines. This was done to rule out IgA deficiency which is common among the patients suffering from CD. Antibodies for detection of this disease are from IgA class; therefore, if IgA deficiency existed, we had to evaluate IgG anti-endomysial antibody.

 In general, serological tests play an important role in diagnosis of CD. In one study, sensitivity and specificity of the IgA anti-tTG was reported as 61-100% (mean: 87%) and 86-100% (mean: 95%), respectively. In addition, 10% of the patients whose disease was distinguished earlier than 2 years of age showed absence of IgA anti-tTG^[^^2^^]^. 

**Duodenal biopsy:**

For a long time, diagnosis of CD was based on the finding of villous atrophy in a biopsy of small intestine. The most relevant feature of the disease was histological change, and histology became the gold standard for diagnosis. Despite substantial changes in the mode of presentation and the availability of new diagnostic tools, small bowel mucosal biopsy is still the gold standard for CD diagnosis^[^^14^^,^^15^^]^. 

 In the present study, biopsy was performed by means of upper gastrointestinal endoscopy (by Olympus vehicle) in case the patients' serum level of IgA was within the normal range (NI) and their anti-tTG level was higher than 18 U/m. In addition, histological examination was done according to Marsh classification^[^^16^^,^^17^^]^. This procedure was done by a pediatric gastroenterologist in this center. The prevalence of CD was calculated based on the proportion of the subjects with positive antibody test results and positive intestinal histology within the sample with a 95% Confidence Interval (95% CI). The patients whose CD had been diagnosed before our investigation, those who expired, and the patients whose parents were not willing to participate in the study were excluded from the research. 

**Statistical analysis:**

All the statistical analyses were performed through the SPSS statistical software (v. 16); t-test was used to compare the case group (the patients with DCM) with the control group. In addition, Pearson correlation coefficient was used to determine the relationship between the variables, such as Anti-tTG, EF, and age. *P*<0.05 was considered as statistically significant.

## Findings

As mentioned before, this study was conducted on 82 subjects, including 41 DCM patients who had been screened for CD and 41 healthy children as the control group. Measured parameter between the patients and controls are shown in Table 1. Although the correlation between EF and Ant-tTG was not statistically significant, a significant difference was observed between the cases (4.44+2.7) and controls (7.02+4.7) regarding the mean Anti-tTG (*P*-value=0.003).

 In this study, IgA deficiency was not detected in any of the patients. In addition, 7/41 (17%) patients had borderline Anti-tTG level among whom 4 (57%) were males and 3 (43%) females; however, the difference was not statistically significant. Moreover, 1 (2.5%) male patient had potentially CD with a high level of Anti-tTG (113) and normal intestinal biopsy. Besides, 33/41 (80.5%) patients had negative screen test implying Anti-tTG level <12.

 In the present study the number of patients which were younger than two years was 8/41 (19.5%) in case and control group. 

**Fig. 1:**
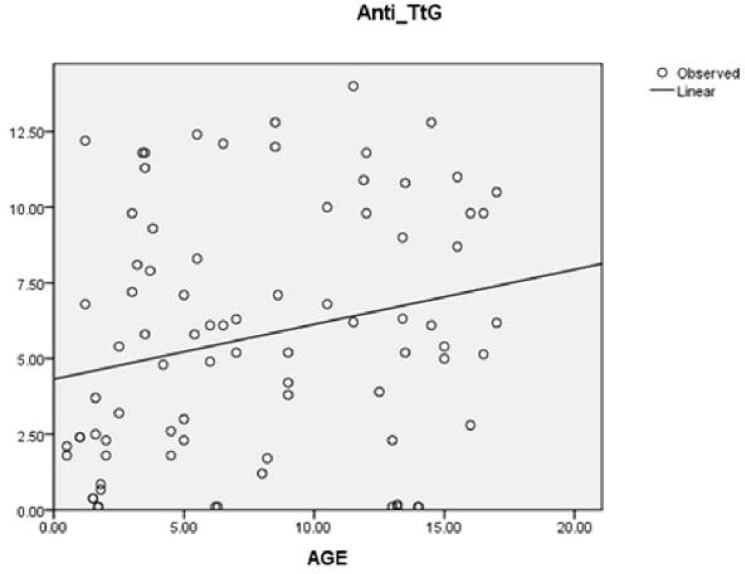
Linear correlation between age and Anti-tTG titer in the children with celiac disease.

 The findings of the current study revealed a linear correlation between age and Anti-tTG titer. This implies that as the children were older, the level of antibody was higher and vice-versa (Fig. 1). 

## Discussion

This study showed that the patients with DCM may have the potential of CD according to serologic tests. The patient with positive anti-tTG serum level and potential CD (prevalence of 2.5%) had no gastrointestinal symptoms (abdominal pain, diarrhea, etc.); therefore, CD was not considered. The patient's DCM had been diagnosed one year ago, with mild (50%) decrease in EF which may not raise a suspicion to CD. According to the previous studies mostly done on adult patients, CD is not infrequent among DCM patients. Moreover, the prevalence of CD in DCM ranges from 1.9% to 5.7% which is important compared to the prevalence rate of 0.4% in the general population^[^^18^^,^^19^^]^.

 Some studies have shown that the CD patients who developed clinical DCM later had early changes on their echocardiograms, which can demonstrate the correlation between these two diseases^[^^5^^,^^20^^]^. 

 The patients with concurrent CD and DCM often are referred to receive medical care regarding signs and symptoms of cardiac problem^[^^21^^]^. Since these signs and symptoms interfere with normal life, they are more obvious for the people.

**Table 1:** Measured parameter between the patients and controls

**Variable**	**Patients** **Mean (SD)**	**Controls** **Mean (SD)**	***P. value***
**Age (year)**	7.7 (5.2)	7.8 (5.2)	0.9
**Weight (Kg)**	23.7 (12.8)	25.5 (12.9)	0.5
**IgA-titer **	1.11 (0.46)	1.23 (0.53)	0.3
**Anti-tissue transglutaminase **	7.02 (4.71)	4.44 (2.74)	0.003

SD: Standard Deviation

 Clinical presentation of CD may be very variable. In general, there are some forms of the disease, such as silent (with positive antibody and biopsy without any clinical manifestations), potential (with positive antibody, normal biopsy, and no signs or symptoms), and latent CD which may develop later^[^^16^^,^^17^^,^^22^^]^ with extradigestive manifestations and/or mild gastrointestinal symptoms, none of which makes us suspicious to CD leading to misdiagnosis. In case these occur together with the signs of Heart Failure (HF), attention is distracted from the gastrointestinal symptoms.

 DCM often changes the intestinal permeability especially when there is cardiac cachexia. Hypoxia which is responsible for the change in the intestinal permeability^[^^21^^]^ may decrease absorption of important micronutrients for myocardial contraction, one of them being carnitine. Carnitine deficiency in CD has been shown in some studies and this can be one of the parameters explaining this co-morbidity^[^^6^^,^^8^^,^^17^^]^. An-other factor is autoimmunity which has been proved to play an important role in both diseases. Autoimmunity may be a probable reason for extra intestinal signs and symptoms of CD, too^[^^23^^,^^24^^]^. One other mechanism can be genetic disturbance as some gene disorders, such as *ERBB2*, are common between these two diseases. 

 All what was mentioned above can reveal the importance of CD screening in the cases with DCM^[^^19^^,^^25^^,^^26^^]^.

 In this study, the serum level of IgA anti-tTG was correlated with age. Antibody level against human**-**tissue-transglutaminase rises when the patient is exposed to gluten. The probability of increase in the antibody level is considered according to age because the more the patient grows up, the more will be the possibility to be exposed to gluten, the responsible agent in CD, which may lead to higher levels of antibody titer^[^^22^^]^. It becomes more obvious if we know 10% of children younger than two years show absence of IgA anti-tTG,^[^^12^^]^. In this study 8/41(19.5%) of cases and 7/41(17%) of controls were of the said age group. Besides, anti tTG in DCM patients with long term hospitalization and total parenteral nutrition may not be exposed to gluten. This important point may explain why antibody levels could not rise remarkably.

 To the best of our knowledge, the present study is the first one performed in children in south Iran. However, the possibility of such a relationship was stated in the previous studies conducted on adults^[^^21^^]^. The findings of the present study indicated a linear relationship between anti-tTG antibody and age. The importance of this finding is revealed by considering that 17% of the patients with borderline antibody level have to be evaluated for CD in future. Of course, further studies are required to be conducted on the issue to confirm this relationship.

 Up to now, few studies have shown CD diagnosis in DCM children. Elfström et al stated that the risk of heart disease in CD patients is higher when the diagnosis is established in adulthood^[^^19^^]^.

 Since longer exposure to gluten seems to be necessary for development of CD and considering the possibility of association between CD and DCM, it seems logical to evaluate the patients with heart disease for CD.

 The few studies performed on children with CD have shown mild cardiac abnormalities in patients who consume gluten. For instance, Polat et al found a decrease in cardiac abnormalities after starting a gluten-free diet^[^^27^^]^. 

 More recently, a study showed that 1.9% of the patients entering the waiting list for heart transplantation for end-stage cardiomyopathy had positive endomysial antibody compared to 0.35% of the healthy subjects^[^^28^^,^^29^^]^.

 According to the present study and the previous researches, screening for CD may have some benefits even in the DCM children with no gastrointestinal symptoms. Gluten-free diet restores the intestinal permeability allowing adequate absorption of the drugs used in HF treatment^[^^27^^]^. Therefore, such diet may minimize the progression of the heart disease and delay the indication for heart transplantation^[^^30^^]^.

 Antibodies are highly sensitive and specific and their identification is a criterion for selection of patients for intestinal histology^[^^6^^]^. In a Brazilian article, this relationship was mostly found with anti-EMA^[^^31^^]^.

 Our study had some limitations. First, DCM is rare among children; thus, gathering enough cases was almost difficult. Moreover, since conducting studies on children requires their parents’ permission, sometimes we were limited by their refusal. Furthermore, no information was available on the cardiologic response of our patients after starting the gluten-free diet.

## Conclusion

In summary, the present study demonstrated that some patients with DCM may have potential for CD according to serologic evaluation. Hence, it is important to investigate CD in all the children with DCM of unknown cause. But this relationship is not approved and more research must be done.
